# Efficacy of repetitive transcranial magnetic stimulation for post-stroke depression: a systematic review and meta-analysis of randomized clinical trials

**DOI:** 10.1590/1414-431X202010010

**Published:** 2021-01-15

**Authors:** D. Shao, Z.N. Zhao, Y.Q. Zhang, X.Y. Zhou, L.B. Zhao, M. Dong, F.H. Xu, Y.J. Xiang, H.Y. Luo

**Affiliations:** 1 The Second Affiliated Hospital of Chongqing Medical University, Yuzhong District, Department of Neurology, Chongging China Department of Neurology, The Second Affiliated Hospital of Chongqing Medical University, Yuzhong District, Chongging, China; 2 The First Affiliated Hospital of Chongqing Medical and Pharmaceutical College, Department of Neurosurgery, Chongqing China Department of Neurosurgery, The First Affiliated Hospital of Chongqing Medical and Pharmaceutical College, Chongqing, China; 3 The First Affiliated Hospital of Chongqing Medical University, Department of Psychiatry, Chongqing China Department of Psychiatry, The First Affiliated Hospital of Chongqing Medical University, Chongqing, China; 4 Yongchuan Hospital of Chongqing Medical University, Department of Neurology, Chongqing China Department of Neurology, Yongchuan Hospital of Chongqing Medical University, Chongqing, China

**Keywords:** Mental disorders, Noninvasive treatment, Stroke, Meta-analysis, Randomized controlled trials

## Abstract

We aimed to conduct a meta-analysis to evaluate the efficacy of repetitive transcranial magnetic stimulation (rTMS) in patients with post-stroke depression (PSD). Six relevant electronic databases (PubMed, CENTRAL, Embase, Web of Science, CINAHL, and PsycINFO) were searched. Randomized controlled trials (RCTs) that compared rTMS with control condition for PSD were included. The mean change in depression symptom scores was defined as the primary efficacy outcome. Secondary outcomes included the remission rate of depression, stroke recovery, and cognitive function recovery. In total, 7 RCTs with 351 participants were included. At post-treatment, rTMS was significantly more effective than the control condition, with a standardized mean difference (SMD) of -1.15 (95%CI: -1.62 to -0.69; P<0.001, I^2^=71%) and remission with an odds ratio (OR) of 3.46 (95%CI: 1.68 to 7.12; P<0.001; I^2^=11%). As for stroke recovery, rTMS was also better than the control condition (SMD=-0.67, 95%CI: -1.02 to -0.32; P<0.001). However, no significant difference was found for cognitive function recovery between the two groups (SMD=4.07, 95%CI: -1.41 to 9.55; P=0.15). To explore the potential moderators for the primary outcome, a series of subgroup and sensitivity analyses were performed. The results implied that rTMS may be more effective in Asian samples than in North American samples (P=0.03). In conclusion, from the current evidence in this study, rTMS could be an effective treatment for patients with PSD. Further clinical studies with larger sample sizes and clearer subgroup definitions are needed to confirm these outcomes.

## Introduction

Stroke is one of the major public health concerns in the world, because of its high morbidity, mortality, and disability rates and serious financial burden (1). As one of the major obstacles in the process of stroke recovery, post-stroke depression (PSD) has attracted more and more attention (2). Studies have shown that PSD is associated with increased physical disabilities, impaired cognitive and social function, poor quality of life, and high risk of stroke recurrence and suicidality (3). Therefore, the treatment for PSD could benefit stroke functional recovery (4).

Several treatment options are now available for PSD, including medications, psychotherapy, and physiotherapy (5–7). Antidepressants are widely used in various somatic diseases with depression (8), but there are many concerns focused on their safety. Due to the fact that many people who suffer from stroke are elderly or middle-aged with multiple risk factors of arteriosclerotic cardiovascular disease, antidepressants are usually cautiously prescribed for these people because of their common side effects such as blood pressure instability and QT interval prolongation (9,10). Some studies have demonstrated the efficacy of psychological therapy for PSD patients (11). However, most people worldwide, especially in low-income countries, cannot access it.

Compared to medication, physiotherapy have been demonstrated to be safer for elderly people. Among the techniques, repetitive transcranial magnetic stimulation (rTMS) is one of the most important means for PSD patients (12). rTMS uses an electromagnetic coil applied to the scalp to produce a magnetic field. It causes cortical neurons in corresponding areas and neurons in distant areas to discharge, thereby regulating the function of the local cortex and related brain networks (13,14).

In the past decades, rTMS has been widely used in the treatment of psychiatric disorders, especially for major depressive disorder (MDD) (15). A number of studies have shown that rTMS is effective and safe for MDD patients (16). The possible mechanism could be a series of physiological effects induced by the focused magnetic field on specific brain regions, increasing the level of brain-derived neurotrophic factors (BDNF) and enhancing glucose metabolism in the cortex and other specific neural networks (17). However, whether rTMS is likewise effective for PSD patients remains unknown. Some studies have demonstrated promising results, but the conclusions are limited by the small sample size (18–20). Therefore, a well-designed meta-analysis to pool the comprehensive clinical evidence is needed to guide the application of rTMS in PSD patients.

## Material and Methods

### Search strategy and selection criteria

Six relevant electronic databases (PubMed, CENTRAL, Embase, Web of Science, CINAHL, and PsycINFO) were searched from each database inception to December 2019, using different combinations of the following key words and their variants: “depression”, “stroke”, “vascular”, and “transcranial magnetic stimulation”. The detailed search strategy is provided in Supplementary Table S1. Additional articles were identified by scanning conference abstracts and reference lists of the articles obtained from the initial search and contacting the authors of the relevant RCTs for more information. The study protocol is available online (https://data.mendeley.com/datasets/kxcvx66s6g/1).

The following inclusion criteria were applied to the literature search: 1) Design: randomized controlled trials; 2) Participants: diagnosis of stroke (confirmed by computed tomography or nuclear magnetic resonance imaging) or have at least three cardiovascular risk factors; meet a diagnosis of depressive disorder according to a standardized diagnostic interview (e.g., Diagnostic and Statistical Manual of Mental Disorders, and Chinese Classification and Diagnostic Criteria of Mental Disorders (CCMD)), or present a current depressive status defined as scoring above a validated cut-off on a depressive rating scale (e.g., HAMD); 3) Intervention and control group: the efficacy of rTMS or combined therapy (rTMS plus antidepressants) was compared with that of a control condition, such as sham condition or usual treatment; and 4) Outcomes: the primary outcome was the mean change in scores from baseline to post-treatment in depressive rating scales. If there were more than one depressive rating scale used in a trial, we preferred the Hamilton Depression Scale (HAMD) as the first choice. Other scales, such as Montgomery Depression Rating Scale (MDRS) and Beck Depression Inventory (BDI), were also used as an alternative. The secondary outcomes included depression remission rate, stroke recovery, cognitive function recovery, and activities of daily living (ADL). The depression remission rate was defined as the proportion of patients whose validated depression rating score was below the determined threshold (e.g., HAMD ≤7). Stroke recovery was assessed by the mean change in scores between baseline and post-treatment in stroke severity or functioning rating scales, such as National Institutes of Health Stroke Scale (NIHSS) (21), and Modified Scandinavian Stroke Scale (22). The cognitive function recovery was measured as the mean change in scores in cognitive rating scales, e.g., Mini-Mental State Examination (MMSE) (23). No language restrictions were applied to our study. Duplicate and secondary analyses studies were excluded.

### Data extraction and quality assessment

Two review authors (D.S. and Y.Z.) independently judged whether the study met the inclusion and exclusion criteria, evaluated the quality of the article, and completed a standardized data abstraction form. Any disagreements were resolved through discussion and sometimes with another reviewer (X.Z.) if necessary. For studies with incomplete data, a contact with the corresponding authors was attempted.

We used the Risk of Bias Assessment Tool from the Cochrane Handbook for Systematic Reviews of Interventions (http://www.training.cochrane.org/handbook) to evaluate the quality of included studies. The risk of bias was assessed and rated as “low risk”, “high risk”, or “unclear risk” according to the following domains: 1) sequence generation; 2) allocation sequence concealment; 3) blinding of participants and personnel; 4) blinding of outcomes assessment; 5) incomplete outcome data; 6) selective outcome reporting; and 7) other potential sources of bias. The criteria for rating the overall risk of bias of a study was as follows: high risk of bias (one or more domains were rated as “high risk”), low risk of bias (all domains were rated as “low risk”), unclear (all other situations). Any disagreements were resolved by consulting a third reviewer (X.Z.).

### Statistical analysis

We performed a pairwise meta-analysis using Review Manager, version 5.3 (Cochrane Information Management System) and Stata version 14.0 (StataCorp, USA). We calculated the pooled estimates using standardized mean difference (SMD) for continuous outcomes and odds ratio (OR) for dichotomous outcomes. Heterogeneity was assessed using the chi-squared test and I-squared index (I^2^). A P value <0.1 was deemed statistically significant, and I^2^ values of 25, 50, and 75% represented low, moderate, and high heterogeneity, respectively. A fixed-effects model was used to analyze the pooled data with low heterogeneity. For moderate or high heterogeneity, a random-effects model was used to analyze the pooled data (24). The potential publication bias was evaluated by a funnel plot.

Considering that different clinical characteristics and intervention parameters may significantly affect treatment outcomes, we performed the following subgroup analysis for the primary outcome: 1) stimulation pattern (bilateral, low frequency *vs* unilateral, high frequency *vs* unilateral low frequency); 2) number of treatment sessions (≥10 *vs* <10); 3) augmentation with antidepressants (augmentation *vs* non-augmentation); 4) depression diagnosis criteria (diagnosed with DSM or CCMD *vs* diagnosed with rating scales); 5) control conditions (sham-rTMS controlled *vs* routine treatment controlled); and 6) region (North America *vs* Asia). In addition, we performed sensitivity analyses by omitting trials that were rated as high risk of bias, or trials of vascular depression. All the tests were bilateral tests, and statistical significance was defined as a probability value of P<0.05.

## Results

### Study selection and characteristics

**Figure 1:**
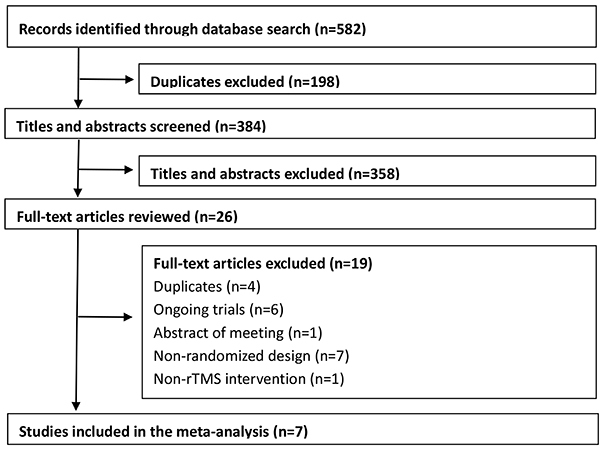
Flow chart of the screening process.

Overall, we found 582 citations from the search (Supplementary Table S2). After excluding duplicates, 384 studies remained. Of these, 358 were excluded based on their titles and abstracts because they did not meet the inclusion criteria. Two review authors independently read the full texts and excluded 19 studies for the following reasons: duplicate (n=4), ongoing trials (n=6), meeting abstract without available data (n=1), non-randomized design (n=7), and no rTMS intervention (n=1). In total, seven trials were included in this meta-analysis (7,1719,). Figure 1 shows a flowchart describing the inclusion process.

**Figure 2:**
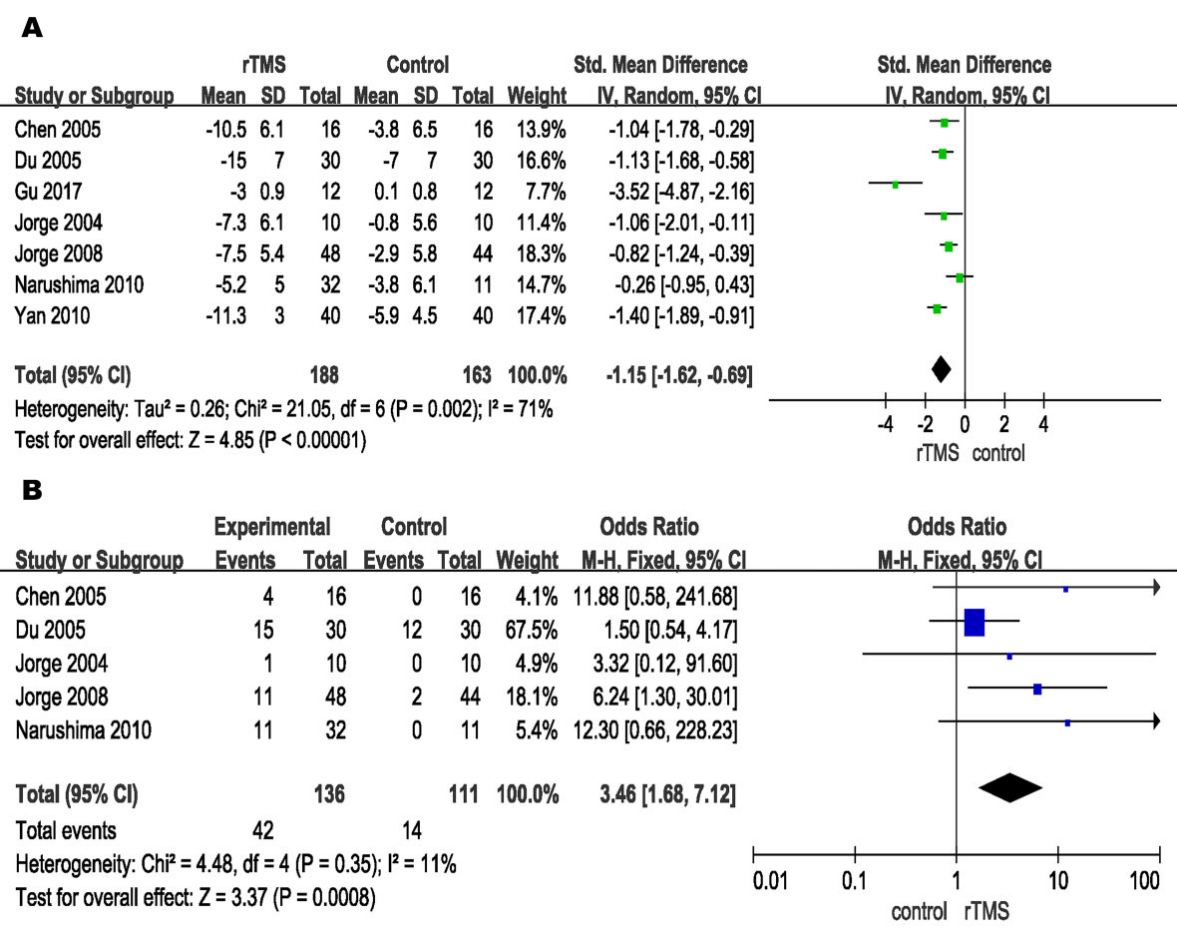
**A**, Comparison of repetitive transcranial magnetic stimulation (rTMS) and control conditions for primary efficacy outcome and **B**, remission of depression in post-stroke depression patients based on change in scores in related rating scales. See Table 1 for corresponding numbers of references.

**Figure 3:**
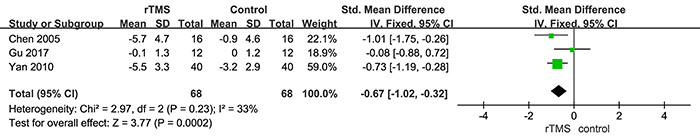
Comparison of repetitive transcranial magnetic stimulation (rTMS) and control conditions for stroke recovery in post-stroke depression patients based on change in scores in related rating scales. See Table 1 for corresponding numbers of references.

**Figure 4:**
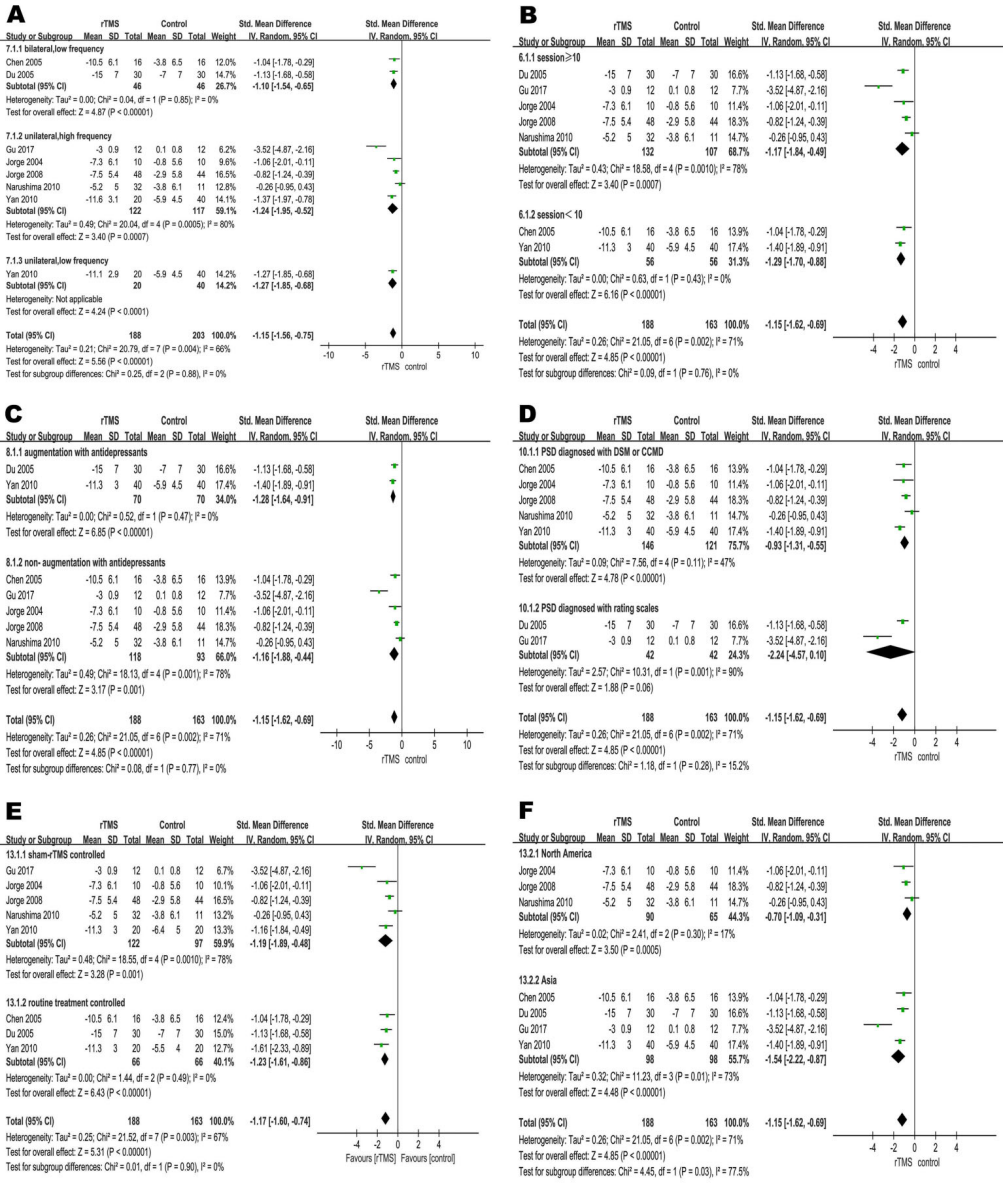
Subgroup analyses of repetitive transcranial magnetic stimulation (rTMS) and control conditions for efficacy. **A**, Forest plot of standardized mean difference (SMD) for change in scores in rating scales for stimulation pattern; **B**, treatment sessions; **C**, augmentation with antidepressants; **D**, depression diagnosis criteria; **E**, for control conditions; **F**, study region. See Table 1 for corresponding numbers of references.

**Figure 5:**
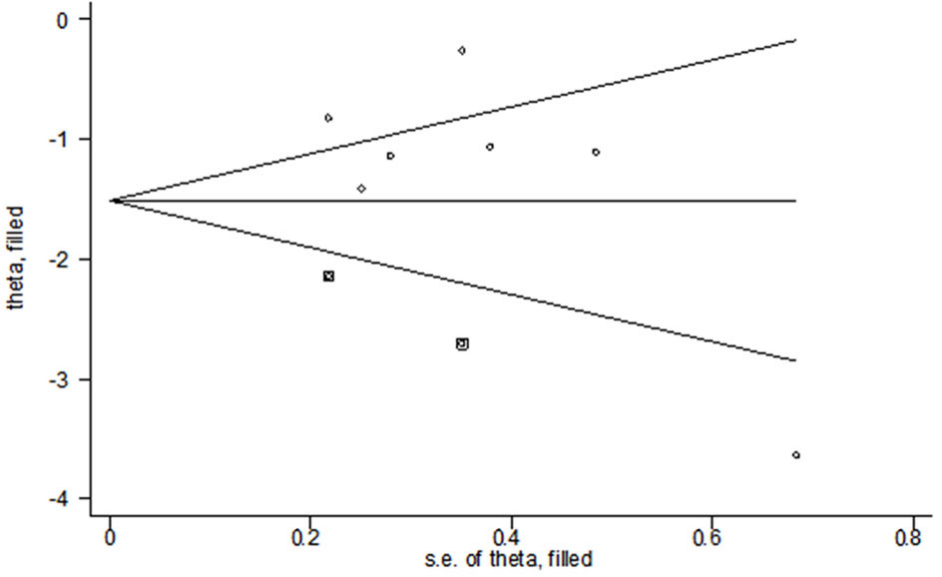
Filled funnel plot of the studies included in the meta-analysis, with pseudo 95% confidence limits.

**Table 1:** Characteristics of randomized clinical trials included in the systematic review about comparison of repetitive transcranial magnetic stimulation (rTMS) and control conditions for post-stroke depression.

Study (year, country, ref.)	Depression (diagnosed criteria)	Mean age±SD (years)	rTMS group	Control group	Duration	Outcome measured
Chen et al. (2005) China (18)	CCMD-2R	61.3±4.7	Bilateral-rTMS+Routine treatment	Routine treatment	1w	HAMD, mSSS, Remission
Du et al. (2005) China (19)	HAMD-17>8	57.6±10.8	Bilateral-rTMS+Fluoxetine+Routine treatment	Fluoxetine+Routine treatment	4w	HAMD, MMSE
Gu and Chang (2017) Korea (20)	BDI>12 and HAMD-17>6	60.8±9.1	Unilateral-rTMS	Sham stimulation	2w	HAMD, MBC
Jorge et al. (2003) USA (7)	DSM-IV	64.8±10.2	Unilateral-rTMS	Sham stimulation	3w	HAMD, Remission
Jorge et al. (2008) USA (25)	DSM-IV	63.7±8.9	Unilateral-rTMS	Sham stimulation	3w	HAMD, Remission
Narushima et al. (2010) USA (26)	DSM-IV	62.8±8.5	Unilateral-rTMS	Sham stimulation	3w	HAMD, MMSE, Remission
Yan et al. (2010) China (27)	CCMD-3R	68.6±7.9	Unilateral-rTMS+Deanxit (left DLPFC, 10Hz,110%RMT)/(right DLPFC,1Hz, 110%RMT)	Sham stimulation+Deanxit	1w	HAMD, NIHSS

CCMD: Chinese Classification and Diagnosis of Mental Diseases; HAMD: Hamilton Depression Scale; BDI: Beck Depression Inventory; DSM: Diagnostic and Statistical Manual of Mental Disorders; DLPFC: dorsolateral prefrontal cortex; Deanxit: Flupentixol and Melitracen tablets; mSSS: modified Scandinavian Stroke Scale; MMSE, Mini-Mental State Examination; MBC: modified Brunnstrom Classification; NIHSS: National Institutes of Health Stroke Scale.

The characteristics of the included trials are described in Table 1. The included RCTs were published between 2004 and 2017. The sample size ranged from 24 to 92 patients per trial, with a median of 50. Overall, 188 participants were randomly assigned to rTMS group and 163 to control group. The mean (SD) age of participants was 63.2 (9.6) years, and approximately half of those patients were male (176, 50.1%). The mean number of sessions was 11.57 (range 7-20) and the mean treatment duration was 2.43 weeks (range 1-4). Participants in two studies were recruited from Asia, and the remaining were from North America. Two RCTs focused on vascular depression, which has a similar pathogenesis to PSD (25,26).

### Efficacy outcomes

The result of the primary efficacy outcome is shown in Figure 2A. The overall pooled effect size showed a significant advantage of rTMS compared with control conditions, with a SMD of -1.15 (P<0.001) and moderate heterogeneity (I^2^=71%; P=0.002). As for the remission rate of depression, patients receiving rTMS were more likely to acquire symptom remission than patients receiving control conditions (OR=3.46, P<0.001; Figure 2B). In terms of the stroke recovery, the rTMS group also was significantly superior to the control conditions (SMD=-0.67, P<0.001, Figure 3). The result of cognitive function recovery showed that there was no significant difference between the two groups (SMD=4.07, P=0.15, Supplementary Figure S1). Since only one trial evaluated the activities of daily living, we were unable to perform a meta-analysis for this outcome.

### Subgroup and sensitivity analyses

We conducted six different subgroup analyses to explore the potential moderators of the primary efficacy outcome. No significant differences were found between the different subgroups in stimulation pattern, number of treatment sessions, augmentation with antidepressants, depression diagnosis criteria, and the different control groups (Figure 4A-E). In the subgroup of different region (Figure 4F), rTMS was more effective in Asian studies than in North American studies (P=0.03). In sensitivity analysis, we did not find a significant change of results from primary efficacy outcome when we omitted trials rated as high risk of bias (SMD=-1.24, P<0.001, Supplementary Figure S2). In view of the difference between post-stroke depression and vascular depression, we conducted a sensitivity analysis by removing the trials focused on vascular depression. The pooled effect was not significantly changed (SMD=-1.44, P<0.001, Supplementary Figure S3). Given the small number of studies, we performed a sensitivity analysis by excluding each single study sequentially. When we excluded the study by Gu and Chang (20), heterogeneity across trials was significantly reduced (I^2^=37%), whereas the pooled estimate was not significantly changed (SMD=-0.97, P<0.001, Supplementary Figure S4).

### Quality assessment and publication bias

Overall, the quality of the included studies in our meta-analysis was low to moderate. Two (28%) trials were rated as high risk of bias, and five (62%) were moderate (details in Supplementary Figure S5A). The risk of bias was rated as high concerning blinding of participants in two RCTs and masking of outcome assessors in one RCT (Supplementary Figure S5B). For the primary efficacy outcome, the funnel plot seemed asymmetric (Supplementary Figure S6), suggesting a potential publication bias. We, therefore, performed a filled funnel plot using the trim-and-fill method. The adjusted SMD was -1.513 (95%CI: -2.056 to -0.971), indicating that rTMS groups were still significantly superior to the control conditions (P<0.001, Figure 5).

## Discussion

In this study, we carried out a meta-analysis to pool the currently best clinical evidence of rTMS for the treatment of PSD. Our meta-analysis found that rTMS was an effective treatment for improving depressive symptoms in patients with PSD, compared with control conditions. Due to data limitations, our study only assessed the improvement of depressive symptoms at the end of treatment, while da Silva Júnior et al. reported that rTMS continued to relieve depressive symptoms until 1 month after the end of the treatment (28). The potential of a long-lasting effect of rTMS still needs to be explored further.

We conducted a series of subgroup analyses to further explore the potential moderators that may influence the result, which included the stimulation pattern, number of treatment sessions, augmentation with antidepressants, depression diagnosed criteria, different control conditions, and study region. The results showed that none but the study region significantly affected the primary outcome, and rTMS may be more effective in Asia than in North America (P=0.03). We considered that the non-blind design in some Asian studies may underestimate the placebo effect. Since the rTMS stimulation parameters such as frequency, intensity of RMT, and coil position vary among different studies, it is difficult to determine which specific parameter is best for clinical practice. For MDD, clinical consensus recommends high-frequency stimulation for the left dorsolateral prefrontal cortex (DLPFC) and low-frequency stimulation for the right DLPFC (29). The pathogenesis of MDD is similar to that of PSD, so we speculated that the selection of stimulation locations mentioned above may also be applicable to PSD. For stimulus intensity, MDD studies recommend 120 motor thresholds (MT) (29), while most PSD studies choose 110 MT (7,20,25-27). A large number of follow-up studies are still needed to explore the appropriate parameters.

Our study conducted three sensitivity analyses. The results of sensitivity analysis by omitting trials within high risk of bias suggested that the efficacy outcomes were robust. The sensitivity analysis after removing the trials focused on vascular depression suggested that adding data on vascular depression did not affect our findings. However, due to the small sample size of this data, this result requires further verification. In the sensitivity analysis by excluding each study one by one, we found that the study by Gu and Chang (20) was the major source of heterogeneity. We considered that it may be related to the small sample size of the study, making the treatment effect exaggerated (30). The results did not change after we excluded the trial (P<0.001).

Some studies have also explored the efficacy of rTMS in the treatment of PSD. Shen et al. (31) included 22 trials and reported that rTMS had a good effect on patients with PSD with a large effect size (mean difference, MD=-6.09), but with a high heterogeneity (I^2^=96%). Compared with our study, Shen et al. searched four Chinese databases and included more Chinese trials. The main reason for not including more Chinese studies was that most are unclear about the description of the clinical process, and after our verification, many of these studies had a case-control design; therefore, we did not search Chinese databases.

In terms of secondary outcomes, our results showed that the remission rate of depression of the rTMS groups was statistically higher than that of the control groups. In terms of stroke recovery, better improvement was found in the rTMS groups than in the control groups. This positive result is consistent with the findings of many studies on the effect of rTMS on stroke patients without depression. In 2012, a multicenter RCT, which included 204 patients with hemiplegia after stroke, found that low-frequency rTMS could promote the recovery of limb function on the hemiplegic side, and there were no adverse reactions in all patients (32). Some studies considered that the effect of rTMS for stroke recovery may be related to its improvement on cortical plasticity and synaptic plasticity in the central nervous system (33). In addition, some studies found that rTMS therapy could also increase the initiative of patients and promote active rehabilitation exercise by improving depressive symptoms, and finally achieve neurological function recovery (34). In our study, one trial reported the results of ADL, which showed that rTMS played a positive role in improving ADL in patients with PSD.

Based on our limited trial data, there was no significant difference in cognitive recovery between the two groups, but with a high degree of heterogeneity. We consider the high heterogeneity may due to two studies and too few participants included in our study. However, many previous studies have shown that rTMS is effective in improving cognitive function of patients without PSD. Anderkova et al. showed that rTMS improves cognitive function of patients with mild cognitive impairment and Alzheimer’s disease (35). Some studies have shown that rTMS can improve the cognitive function of patients by regulating cortical excitability, improving cerebral blood flow and cerebral metabolism, affecting the transmission of various neurotransmitters and the level of gene expression (36).

Safety is another important index for evaluating a treatment method. In the present study, there were two trials that described adverse events, including transient headaches, local discomfort at the site of the stimulation, and exacerbation of initial insomnia. Since the sample size was small, no statistical analysis was carried out. rTMS has been widely proven to be safe and tolerable. However, some studies have reported that it has side effects of inducing epilepsy in healthy people, and in depressed patients, tinnitus patients, and patients with chronic pain, all of which were case reports (37). In our study, there was no induction of seizure during the course of treatment, and no patient withdrew from the studies due to adverse events. Therefore, according to the limited evidence, we consider that the side effects of rTMS are slight and controllable.

Our research and previous studies have demonstrated the efficacy of rTMS in the treatment of PSD, but the detailed mechanism is still unclear. Studies have indicated that individuals with PSD may have altered brain network structures, as well as abnormal white matter function. rTMS may reduce depressive symptoms by increasing the fractional anisotropy value in patients with PSD. However, the stimulation target and specific parameters need to be optimized (38). In addition, there are some known pathophysiological mechanisms underlying rTMS in the treatment of PSD, such as increased concentration of BDNF, increased glucose metabolism in the cortex and in specific neural networks, increased neurogenesis, modulation of neurobiochemical effects, and regulation of emotion through long-term potentiation- and long-term depression-like plasticity (39). Although the specific mechanisms underlying the effect of rTMS on PSD are unknown, its effectiveness cannot be denied. Understanding how rTMS affects PSD should help the development of new and more effective treatments, and the mechanism should be further studied to provide a strong theoretical basis for clinical application.

There were some limitations of this meta-analysis. First, this study included a small sample size with a limited source of participants, which may affect the stability of the results. Second, some of the included studies did not clearly describe the allocation sequence concealment and sequence generation, which may be a potential risk of bias for the final results. Third, there was a potential publication bias in this article, and although we have made corrections, it may still have an impact on the results. Fourth, the evidence for PSD is very limited, given the small number of studies and the small number of participants included in each study. There was not enough data to explore the adequate parameters (stimulation site, intensity, and frequency) of rTMS in treating PSD. Fifth, patients with vascular depression were also included in our study, and the clinical characteristics of these patients were different from those of patients with PSD. Although we have performed a sensitivity analysis and the primary result did not change significantly, the inclusion of such patients may still cause risk of bias in the results. Finally, due to the limitations of meta-analysis, individual data and basic clinical information cannot be directly analyzed.

### Conclusions

Repetitive TMS was effective in improving depressive symptoms and promoting stroke recovery. In addition, the side effects of rTMS treatment were minimal and controllable. More research with large sample sizes are needed to confirm the result.

## Supplementary Material

Click here to view [pdf].
